# Hepatectomy and intrahepatic hepaticocutaneous jejunostomy for bilateral primary hepatolithiasis: Case report

**DOI:** 10.1016/j.ijscr.2020.05.051

**Published:** 2020-05-30

**Authors:** André Luís Conde Watanabe, Alexandre Coutinho Teixeira de Freitas, Lucinei Stadnik, Júlio Cezar Uili Coelho

**Affiliations:** Division of Gastrointestinal Surgery, Hospital de Clínicas, Federal University of Paraná, Curitiba, PR, Brazil

**Keywords:** Liver, Lithiasis, Hepatectomy, Case report

## Abstract

**Introduction:**

Primary hepatolithiasis is a rare disease in western countries and it is associated with repeated attacks of acute cholangitis. Without proper treatment, hepatolithiasis can lead to progressive biliary strictures and secondary biliary cirrhosis.

**Presentation of case:**

A 40 years old male was admitted due to recurrent cholangitis during the last 18 years. Bilateral primary hepatolithiasis was diagnosed and treated by left hepatectomy with an intrahepatic hepaticocutaneous jejunostomy. There were no postoperative complications and the patient was discharged after 7 days.

**Discussion:**

The management of patients with primary hepatolithiasis remains a challenging task due to the high incidence of residual and recurrent stones after treatment.

**Conclusion:**

Primary bilateral hepatolithiasis is a complex disease that can be managed with partial hepatectomy with an intrahepatic hepaticocutaneous jejunostomy.

## Highlights

• Primary hepatolithiasis is a rare disease in western countries.
• Primary intrahepatic stones are associated with repeated attacks of acute cholangitis.
• Primary bilateral hepatolithiasis can be managed with partial hepatectomy with an intrahepatic hepaticocutaneous jejunostomy.

## 1 Introduction

Hepatolithiasis is a common disease in East Asia. In Taiwan, the relative incidence has been reported to be 20% of all gallstone diseases [1]. However, it is rare in western countries, where the incidence varies from 0.6% to 1.3% [2,3]. In Brazil, primary hepatolithiasis accounts for 2.1% of all patients with gallstone diseases admitted for treatment in a tertiary center [4].

Primary intrahepatic stones are associated with repeated attacks of acute cholangitis. Without proper treatment, hepatolithiasis can lead to progressive biliary strictures, liver abscess, atrophy of the parenchyma and secondary biliary cirrhosis. Furthermore, the association between hepatolithiasis and cholangiocarcinoma is well recognized. In recent series, the incidence of cholangiocarcinoma in patients with hepatolithiasis has been reported to be 10–12% [5,6].

Currently, hepatic resection and percutaneous transhepatic cholangioscopic lithotomy (PTCSL) are the main approaches to the treatment of hepatolithiasis [7,8]. Partial hepatectomy is considered the most definitive treatment for hepatolithiasis as it removes the intrahepatic stones, the strictured bile ducts and the affected liver segments, which harbor bacteria and serve as a source of infection. Long-term follow-up of partial hepatectomy for hepatolithiasis has shown a high immediate stone clearance and a low stone recurrence rates [5,6,8]. However, these studies mainly included patients with unilateral disease. Recently, Yang et al. concluded that partial hepatectomy was a safe and effective treatment in patients with biliary strictures and bilateral hepatolithiasis [9].

The authors report a case of bilateral primary hepatolithiasis treated by left hepatectomy with an intrahepatic hepaticocutaneous jejunostomy. This case is reported in line with the SCARE criteria [10].

## 2 Presentation of case

A 40-year-old man was referred to our institution with an 18-year history of recurrent episodes of epigastric pain, jaundice and fever. Prior to the admission, the patient had undergone an emergency open cholecystectomy due to acute cholecystitis. One day after surgery, a computed tomography scan revealed a diffuse dilation of intrahepatic bile ducts and intrahepatic stones in both lobes of the liver. The patient was then referred to our hospital for further investigation and treatment. There were no other associated diseases.

**Fig. 1:**
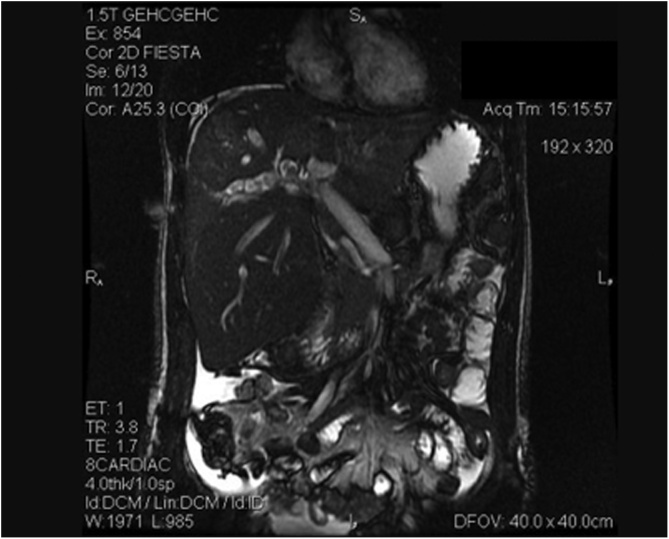
Preoperative MRI showing dilated intrahepatic bile ducts with stones inside.

On admission, the patient complained of right upper quadrant (RUQ) pain and fever. Physical examination revealed jaundice and tenderness over the RUQ area without a palpable abdominal mass. Results of laboratory tests were as follows: hemoglobin: 13.5 g/dL; white blood cell count: 17570/mm^3^; platelet: 421000/μL; international normalized ratio (INR): 1.35; albumin: 2.4 g/dL; total bilirubin: 8.73 mg/dL; direct bilirubin: 6.19 mg/dL; aspartate aminotransferase (AST): 30 IU/L; alanine aminotransferase (ALT): 25 IU/L; alkaline phosphatase (ALP): 372 U/L; blood urea nitrogen: 17 mg/dL; creatinine: 0.5 mg/dL; sodium: 137 meq/L; potassium: 3.3 meq/L. The diagnosis of acute cholangitis was considered and a broad spectrum antibiotic was started. Subsequent abdominal MRI showed hepatomegaly with diffuse dilated intrahepatic ducts (Fig. 1). Intrahepatic stones, biliary strictures and hepatic abscesses were identified in both lobes of the liver, especially in the left segments.

**Fig. 2:**
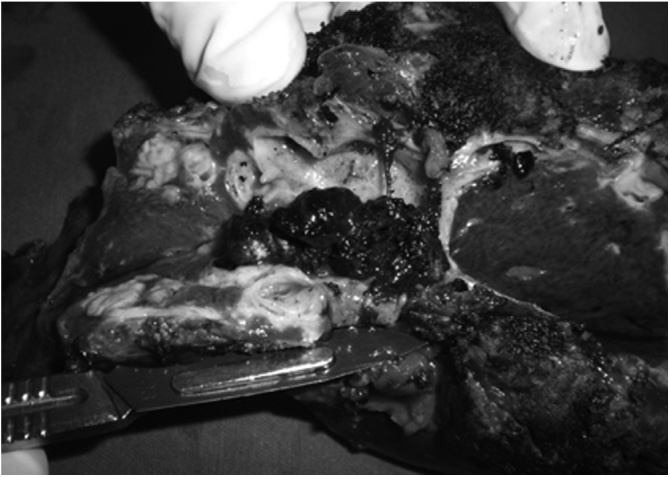
Left lobe parenchyma presenting dilated bile ducts with multiple intrahepatic stones.

After complete remission of the acute cholangitis-related symptoms, a left hepatectomy with an intrahepatic Roux-en-Y hepaticocutaneous jejunostomy was performed. Intraoperative assessment showed the duodenum firmly adhered to the hepatic hilum, probably due to the previous open cholecystectomy. During the parenchyma dissection, multiple stones were observed inside the dilated bile ducts. Fig. 2 shows these findings during specimen analysis. The residual stones in the right lobe were removed by saline flushing through the opened bile ducts in the parenchyma. In order to allow endoscopic access for the removal of recurrent stones, a hepaticocutaneous jejunostomy was performed. This procedure consisted of lengthening the Roux limb of the hepaticojejunostomy to the cutaneous level, which was buried subcutaneously in the anterior abdominal wall to facilitate later access to the biliary tree to remove possible recurrent stones. The postoperative course was uneventful and the patient was discharged on the 7th postoperative day. Pathological examination revealed intense chronic and acute cholangitis without malignant lesions.

## 3 Discussion

The management of patients with primary hepatolithiasis remains a challenging task. The definitive treatment includes the complete removal of the stones and the establishment of a satisfactory drainage to the obstructed biliary system in order to prevent future attacks of cholangitis and to avoid progression to secondary biliary cirrhosis. Frequently, multiple surgical procedures are performed before referring the patient to a specialized center. In recent studies, 40–47% of patients with hepatolithiasis had previous biliary surgery, including cholecystectomy as the most common procedure [5,6,9]. As seen in our case, these previous operations are associated with intra-abdominal adhesions which certainly complicate the definitive surgery.

Hepatectomy is the most effective treatment for hepatolithiasis and has traditionally been used for unilateral disease. However, patients with bilateral hepatolithiasis are more complicated and difficult to manage [9]. There is a high incidence of residual stones in these patients, regardless of the treatment method [7]. Furthermore, they require more radiologic studies, clinic visits and hospital admissions as compared to patients with unilateral disease [11]. Chen et al. demonstrated that patients with bilateral hepatolithiasis would benefit from hepatic resection of the more severely affected side combined with hepaticocutaneous jejunostomy for contralateral latter stones removal [5]. This is the same approach that we adopted in our case. In a series including 136 patients with biliary strictures and bilateral hepatolithiasis, Yang et al. evaluated the efficacy of bilateral and unilateral hepatectomy [9]. In this study, the bilateral and the unilateral hepatectomy groups had comparable results, with 5-year survival rates of 98% and 91.5%, respectively [9]. The authors concluded that in this selected group of patients with bilateral hepatolithiasis, the extent of partial hepatectomy should be balanced between the extent of intrahepatic stones and ductal strictures and the liver functional reserve.

Residual and recurrent stones are the main problems after treatment for hepatolithiasis [12]. Intrahepatic biliary stricture is a major cause of treatment failure and stone recurrence [6,7]. In a study including 48 patients with hepatolithiasis treated by PTCSL, Jan et al. reported a stone recurrence rate of 51.6% in patients with biliary stricture, whereas no recurrence was seen in patients without stricture [13]. Hepatic resection can reduce the risk of recurrent stone formation, since it removes not only the intrahepatic stones but also the strictured bile ducts [5]. In addition, a hepaticocutaneous jejunostomy was also performed in our patient. This procedure offers the advantage of a permanent percutaneous access to the biliary tree allowing removal of recurrent stones and dilatation of strictures without the need of further surgery [14].

## 4 Conclusion

Primary hepatolithiasis is a rare disease in western countries, including in Brazil. The optimal management of these patients includes a complete evaluation and complex procedures and should be done in specialized centers. We reported a case of bilateral primary hepatolithiasis successfully treated by partial hepatectomy with an intrahepatic hepaticocutaneous jejunostomy.

## Declaration of Competing Interest

Nothing to declare.

## Sources of funding

Nothing to declare.

## Ethical approval

It is a case report of a technique already described in the literature exempt from an ethnical approval in our institution.

## Consent

A fully informed written consent was obtained from the patient.

## Author contribution

**André Watanabe:** study concept, data collection, writing the paper. **Lucinei Stadnik:** data collection. **Alexandre Freitas:** supervision, review. Júlio Cezar U. Coelho: supervision, review and editing.

## Registration of research studies

1. Name of the registry: Not applicable.
2. Unique identifying number or registration ID.
3. Hyperlink to your specific registration (must be publicly accessible and will be checked).

## Guarantor

André Watanabe.

## Provenance and peer review

Not commissioned, externally peer-reviewed.
